# Face Masks in Young Children During the COVID-19 Pandemic: Parents' and Pediatricians' Point of View

**DOI:** 10.3389/fped.2021.676718

**Published:** 2021-06-23

**Authors:** Rémy Assathiany, Catherine Salinier, Stéphane Béchet, Claire Dolard, Fabienne Kochert, Alain Bocquet, Corinne Levy

**Affiliations:** ^1^Association Française de Pédiatrie Ambulatoire, Orléans, France; ^2^Association Clinique et Thérapeutique Infantile du Val de Marne, Créteil, France; ^3^Doola, Bordeaux, France; ^4^Clinical Research Center, CHI Creteil, Créteil, France; ^5^Paris Est University, IMRB-GRC GEMINI, Créteil, France; ^6^GPIP, Groupe de Pathologie Infectieuse Pédiatrique, Paris, France

**Keywords:** face mask, pandemic, COVID-19, children, parenthood

## Abstract

**Background:** In countries with high SARS-CoV-2 circulation, the pandemic has presented many challenges on different fronts, affecting lives and livelihoods; efforts to keep schools open are among the most important. In France, to keep schools open, wearing a face mask has been mandatory for children from age 6 years since November 2020.

**Objective:** To evaluate the acceptability and tolerance of this measure by children as well as both parents and pediatricians.

**Setting:** Parents registered on the website of the French Association of Ambulatory Pediatrics and pediatricians members of this association.

**Participants:** All parents and pediatricians who agreed to take part in the survey.

**Results:** Among the 2,954 questionnaires for the parents' survey, the reasons for wearing a mask were understood by 54.6% of parents, most of whom (84.6%) explained the reasons to their children. The parents applied this measure because it was mandatory (93.4%) even if they disagreed (63.3%). When interviewed by parents, children said they were usually embarrassed (80.9%) by the mask. The main symptoms or changes of behavior attributed to the mask according to parents were headache (49.0%), speaking difficulties (45%), change in mood (45.2%) and breathing discomfort (28.1%). Among the 663 pediatricians who responded, many agreed with mandatory mask-wearing at age 6 years (67.7%). Overall, 15% of pediatricians systematically asked about the mask tolerance during the consultation. During the medical consultation, when the parents complained about the mask (64.3%), the main drawbacks were related to fog on glasses (reported by 68.2% of pediatricians), breathing discomfort (53.1% of pediatricians), cutaneous disorders (42.4% of pediatricians) and headaches (38.2% of pediatricians).

**Conclusion:** Despite the many inconveniences reported, children agree to wear the mask better than their parents think. Pediatricians should sufficiently take the opportunity during the consultation to further explain the reasons for wearing the mask because their pedagogical role is crucial.

## Introduction

COVID-19 is an extremely contagious viral disease, with essentially respiratory tropism, due to the SARS-CoV-2 virus. Since December 2019, this virus was responsible for a pandemic that reached more than 100 million people and caused 2.3 million deaths worldwide as of February 2021^1^. Children are less often affected than adults and have exceptionally severe illness (1–4). At the beginning of the pandemic, the disease was assumed to be transmitted mainly via children, as observed during influenza epidemics; school closures proved effective in limiting the spread of the disease (5). By analogy with the flu, to protect children and fight against the spread of COVID-19, as part of the lockdown, many countries made the drastic decision to close schools and universities^2^. Thus, at the end of March 2020, 166 countries had implemented this measure, depriving 1.5 billion schoolchildren and students of education^2^.

However, the effectiveness of this radical school closure measure to fight the pandemic and decrease the risk of contamination of adults working at schools has not yet been demonstrated (6–8). Conversely, the fight against the pandemic has been effective in some countries such as Taiwan, which did not implement widespread school closures (9). In a recent French study, more than 80% of children with positive SARS-CoV-2 results by RT-PCR or serology had a confirmed or suspected COVID-19 household contact (10). Indeed, the most frequent source of contamination of children is not the school but rather the family and home environment (11, 12). Nevertheless, the contamination at school exists but is minor (13, 14). Moreover, the prolonged closure of schools has resulted in adverse consequences for both children and their parents who work from home (15, 16). All of these drawbacks were even greater in low-income families (17). In this context, France made many efforts to keep schools open even with high viral circulation^3^. Until high COVID-19 vaccination coverage is reached with a vaccinated population of all ages, other effective measures should be implemented to limit the spread of the epidemic among children who are often asymptomatic carriers.

Among these measures to limit the spread, maintaining a distance of more than 1 m as long as possible, frequent hand washing, and wearing a face mask have shown their effectiveness (18, 19). Medical or surgical masks help protect people around children and offer some protection to children. The FFP2 (N95) masks that filter 94% of the particles prevents the transmission of the virus from the patient to caregivers but is not suitable for children, except in special cases (e.g., cystic fibrosis or severely immunocompromised children). The visor is useful only if it is used in addition to a mask; it is rarely used by children^4^.

Therefore, in France, to keep schools open, wearing a face mask has been mandatory in middle school since September 2020^5^. During the second lockdown, on November 2, 2020, this requirement was extended to children from age 6 years^5^.

In this context, we surveyed both parents and pediatricians to evaluate the acceptability and tolerance of mask-wearing in young children.

## Methods

The French Association of Ambulatory Pediatrics (AFPA) administered two surveys via their websites (www.mpedia.fr and https://afpa.org/). The two different anonymous questionnaires with closed questions (Supplementary Material) were available online; one was targeted to parents, the other to pediatricians. The data were collected by using SurveyMonkey (SurveyMonkey Inc.).

### Survey Targeted to The Parents

A first group of questions focused on the understanding and acceptance of mask-wearing by parents and children. A second group of questions asked about side-effects possibly attributed to the mask, with a list provided. The last questions asked about the socio-economic profile of the family.

Several e-mails with a link to the SurveyMonkey questionnaire were sent to the 10,000 subscribers of the mpedia.fr site in December 2020 and to different social networks. Parents were asked to answer only if they had a child attending primary school, regardless of age. If there were several siblings, the parents had to answer for the youngest child only.

### Survey Targeted to The Pediatricians

The questions first focused on the demographic characteristics of the pediatrician, followed by their point of view regarding the mandatory requirement for children to wear a face mask. Another group of questions asked about discussions regarding mask-wearing and its possible side-effects between the pediatrician, the child and the parents during a consultation. The last group of questions asked about how the pediatrician could convince both parents and children of the benefits of mask-wearing. Three successive emails with a link to the SurveyMonkey questionnaire were sent to the 1,613 AFPA subscribers in December 2020.

Our data being strictly anonymous, no ethics committee approval was requested for this study.

### Statistical Analysis

Our sample of responding parents included a high number with high socio-professional categories as compared with the 2016 National Perinatal Survey (NPS) study, which is a representative reference of the population of parents of young children living in metropolitan France. For better representativeness, the results of the parent survey were adjusted according to socio-professional situation of the NPS study (20). An adapted coefficient was applied to the parents' responses according to the under/over-represented occupations. This coefficient was defined by the relative frequency of occupation categories according to the parents' survey and the 2016 NPS baseline data. Thus, regarding socio-professional categories, the results obtained can be considered representative of the population of parents with young children living in France. In the absence of a response for the father's occupation, the mother's occupation was used. If the parents' occupation was not given, the questionnaire was not considered.

Responses (frequency and percentage) were analyzed by using Stata/SE v15 (StataCorp, College Station, TX, USA). Chi-square test was used for inter-group comparisons, and *p* < 0.05 was considered statistically significant.

## Results

### Survey Targeted to Parents

Among the 2,954 analyzed questionnaires, the age of the children for whom parents responded was distributed as follows: <6 years (25.7%), 6 years (6%), 7 years (23.9%), 8 years (17.4%), 9 years (10.7%), 10 years (6.8%), and ≥11 years (9.5%). Most of the responses were given by mothers (89.2%) from 30 to 39 years old (54.2%) with often a bachelor's degree (74.5%). All regions in France were represented, with the least responses from Corsica (0.2% of responses) and the most from Auvergne-Rhône-Alpes (17.5% of responses).

#### Understanding and Acceptance of The Mask by Parents and Children

Slightly more than half of the parents (54.6%) said they understood the reasons for wearing a mask; most (84.6%) explained the reasons to their children. Most often, the parents applied this measure because it was mandatory (93.4%) even if they disagreed (63.3%) or were unable to know if it was useful (13.7%). In total, 76.2% of parents stated that their children understood the reasons for wearing a mask, and for many (59.7%), the children had become accustomed to wearing it. Children interviewed by their parents said they were usually embarrassed (80.9%) by the mask. Outside of school, 24.8% of children continued to wear the mask even if it was not mandatory. Globally, according to parents' declarations, accepting the mask was more difficult for children aged 10 years than 6 years (50.8 vs. 23.9%, *p* < 0.001), as was understanding the reasons for wearing it (29.2 vs. 14.7%, *p* = 0.002).

#### Potential Side-Effects of Face-Masking

**Figure 1:**
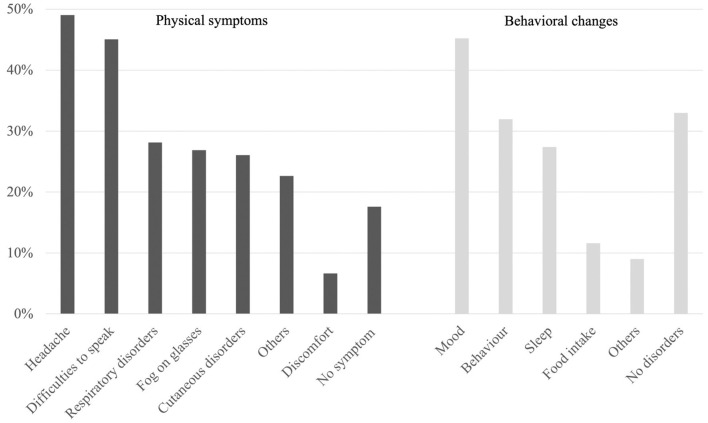
Physical symptoms and behavioral changes attributed to mask-wearing (parents' survey).

Since wearing the mask became a requirement, parents reported several side-effects in children: 82.4% presented different physical symptoms and 67.0% behavioral changes (Figure 1). The main symptoms attributed to the mask by the parents were headache (49.0%) followed by speaking difficulties (45.1%) and breathing discomfort (28.1%). The main changes in behavior reported by the parents were change in mood (45.2%).

When parents or children did not understand the reasons for wearing the mask, child tolerance was reduced, symptoms and behavioral changes were twice as frequent (except fogged glasses), and the number of children who were used to the mask was reduced.

### Survey Targeted to The Pediatrician

Among the 663 pediatricians who replied to the questionnaire, 83.6% were women; 24.7% were <40 years old and 28.0% were >61 years old. All France regions were represented, with the least responses from Corsica (0.5% of responses) and the most from the Paris area (23.0% of responses).

### Pediatricians' Point of View and Discussion With Parents and Children Regarding Mandatory Masks

Many pediatricians agreed with mandatory mask-wearing from age 6 years (67.7%). During the pediatrician consultation, the question of the mask was spontaneously addressed by the parents and/or children (80.9% of pediatricians), and 15.0% of pediatricians systematically asked the question about mask tolerance. According to 23.5% of pediatricians, parents came for a specific visit due to symptoms attributed to the mask. For 64.3% of pediatricians, the parents complained about their child wearing a mask. The main drawbacks were related to fog on glasses (reported by 68.2% of pediatricians), breathing discomfort (53.1% of pediatricians), cutaneous disorders (42.4% of pediatricians) and headaches (38.2% of pediatricians).

### The Convincing Power of The Pediatrician

More than one third of pediatricians (37.1%) reported that a few parents tried to obtain a certificate to justify that their child could not wear the mask, but more than half of these pediatricians (55.7%) convinced the parents. When the reasons for exemption from wearing a mask were justified (33.8% of pediatricians), it was most often for neurodevelopmental disorders (72.3%) and less often for asthma (25.8%).

## Discussion

This study combines the views of pediatricians, parents and therefore their children on the important issue of mask-wearing from age 6 years. Many pediatricians agreed with mandatory mask-wearing from age 6 years (67.7%), but many parents disagreed (63.3%). By contrast to young adults, children in our study agreed to wear the mask with good acceptability (21), and for 59.7% of parents, their children had become accustomed to wearing it. Because they have a significant educational role, pediatricians should take the opportunity during the consultation to further explain the reasons for wearing the mask.

Surprisingly, according to the parents' declarations, children aged 10 years had more difficulties accepting the mask and understanding the reasons for wearing it than children aged 6 years. This result suggests that parents or teachers took more time to explain the recommendations and the reasons to the youngest children. Another possibility is that at age 10, children start to be in pre-adolescence and object to instructions.

According to the parents' and pediatricians' survey, complaints about mask-wearing were common. Indeed, the symptoms attributed to the mask were frequent (82.4%), and headache was reported for almost half of the children. Unfortunately, our questionnaire did not ask whether children previously had headache. Although the prevalence of headache appeared high, it was comparable to that found in children before the pandemic: 37–51% in children >7 years old (22). A similar prevalence of headache (50%) was found in nurses equipped with an FFP2 mask (23). The mechanisms evoked were often multifactorial: mechanical factors, hypoxia, hypercapnia, and stress (24). Another complaint was the children's discomfort in speaking, reported by 45% of parents. This result was not surprising because obviously the mask is a hindrance to elocution. Apart from speech, the mask also hinders non-verbal communication by facial expressions and lip-reading; the understanding of the other is disturbed, and thus interactions are diminished. Moreover, the mask disturbs facial recognition and identification, which is an additional inconvenience for children at school. The facial expression is concealed, so the presence of a possible smile is invisible and the reading of positive or negative emotions is difficult (25). In addition, the teacher's voice is muffled and distorted, which can affect students' comprehension, especially with the presence of significant background noise in the classroom. We did not ask about hearing impairment, although it was often mentioned in the free answers. All of these phenomena are known to contribute to the disruption of teaching and learning at school, especially for children with pre-existing difficulties.

According to parents, just over one quarter of the children in our study experienced breathing discomfort attributed to the mask. Our questionnaire did not collect whether this discomfort occurred in a child sitting at a desk or during exertion in the playground. These difficulties were frequent (36%) in a student population observed in Poland (21). Studies conducted among adults were most often reassuring; in one study, after 1 h of normal walking with an FFP2 mask, no variations were found in pulmonary tidal volume and respiratory rate (26). Also, wearing a mask did not cause significant variation in PO2 and PCO2 at rest or during sedentary activity (27). During muscular exercise, the results were contradictory: measurements taken during exercise in adults with face masks did not show changes in PO2, muscle oxygenation, or heart rate (28). However, as a precaution, the WHO advises against the use of masks during sports activities (29).

Overall, 27% of parents of children wearing a mask reported visual discomfort caused by fogged glasses. The prevalence of children with glasses in fifth grade is 25%^6^; therefore, fogging impairs the vision of all children who wear glasses. The annoying presence of fogging on glasses was also found in a population of students with a similar prevalence (21). Fogging can be avoided by washing glasses with soapy water (30).

Approximately one-quarter of parents reported in their children cutaneous disorders attributed to mask use. Sweat, saliva, and moisture between the mouth and mask were likely responsible. Erythema of the nose, cheeks, and ears was even more common among nurses wearing surgical masks, but the context was different, including 24 consecutive hours of work (23).

More than two thirds of the children had functional problems related to mask-wearing. We can assume that these disorders were more likely related to the anxious and stressful period of the pandemic than mask-wearing itself. Mood disorders (anxiety, sadness, anguish) were the most frequently mentioned and were present in about half of the children. All factors combined can lead to worry in children: children were afraid of being infected; they constantly heard alarming information broadcast by audio-visual media; they also perceived conversations from their parents worried about the situation and about family members' health, especially grandparents who were at risk. Extra-curricular activities were interrupted and relationships and interactions with peers were diminished. Unrecognized and unheeded by parents, temper tantrums or anger can occur and were seen in almost one-third of children. In a study in Shaanxi Province, China, the most common disorders observed in children during the pandemic were irritability, inattention, agitation, and separation anxiety (31). Sleep disturbances were frequently cited by parents and were twice as frequent as compared with a prevalence of 12–17% in children aged 6–12 years (32). Sleep disorders can in turn lead to increased anxiety, mood disorders, learning difficulties, and eating disorders. Only 11% of parents mentioned eating disorders, anorexia or bulimia. In times of stress, there is an increase in eating food, especially sugary foods, decreased outdoor activity, and increased screen use by children, the association being responsible for excessive weight gain (33).

Our study has several limitations. The first is the questionnaire itself, which consisted of a list of symptoms offered to parents and pediatricians. Even if respondents had the option of adding more symptoms, it was easier to check off those previously offered in the questionnaire. However, the symptoms we proposed were those most often reported in the literature. The second limitation was the absence of information regarding the intensity and severity of the symptoms. However, these symptoms rarely led to additional physician consultations, which suggests that they were not usual. Another limitation was the overrepresentation of women who answered both the parents' and pediatricians' surveys. This finding agrees with the distribution of women in the pediatrician medical occupation. Besides these limitations, our study highlights the importance of thinking about accommodations compatible with a comfortable life for children at school and facilitating their learning in the best conditions. Here we propose some advice for pediatricians, parents, children and teachers to promote and accept wearing a mask (Table 1).

**Table 1:** Advice for parents/children, teachers and pediatricians to promote and accept wearing a mask (from 34).

**Recommendations to parents**
Masks are made to protect children and their loved ones
Mask-wearing is a reflex, such as seat belt in a car and helmet on a bicycle
Fit the mask to the child's size
Let the child choose the mask she/he likes
Always have a mask available
Take your time to explain mask-wearing with age-appropriate words
Have the child put on a mask at home, with the parents, to get used to it
Put a mask on a stuffed animal
Parents should be aware of how facial masks can harm the intensity and quality of speech and how much this can affect the school performance of their child
Be aware of the child's daily school performance
Talk to the child about the day at school and about the difficulties he/she may have
Observe behavior changes that may indicate school difficulties
**Recommendations to teachers**
Speak slowly and articulate speech
Use features and visual support and images in activities
Reduce environmental noise and keep the child's attention before speaking
Consider using a portable microphone
Ask the child to repeat the instructions received, making sure that the child has really understood
Repeat the instructions or rephrase your speech if the child is not understanding what is being requested
Do not speak loudly; do not overemphasize, or exaggerate your words
Do not talk to the child while walking; always make eye contact
Avoid using flashy masks because they can compete for the child's attention, dispersing the listener's focus
**Recommendations to pediatricians**
Take advantage of each consultation for talking about the mask and the pandemic, for explaining and detecting side effects or behavioral changes, and for reassuring

## Conclusions

While waiting for effective and widespread vaccination, wearing a face mask is one of the main barrier measures against COVID-19. To avoid further closure of schools, with deleterious consequences, mask-wearing by children is necessary, even if children do not seem highly contagious. Our study showed a moderate adherence of parents to mask-wearing, which was better in children. The side-effects noted by parents were frequent, even if often benign. Of note, the side-effects were significantly reduced when parents adhered to the mask-wearing measure. Therefore, parents must be motivated by constantly renewing the explanations to their children and the justification for this strategy.

## Data Availability Statement

The raw data supporting the conclusions of this article will be made available by the authors, without undue reservation.

## Author Contributions

RA, CS, CL, FK, and CD conceived the study, analyzed the data, and wrote the manuscript. SB, CL, and AB performed the statistical analysis. CL, RA, CS, AB, and SB contributed to writing the manuscript. All authors read and approved the final manuscript.

## Conflict of Interest

The authors declare that the research was conducted in the absence of any commercial or financial relationships that could be construed as a potential conflict of interest.

## Footnotes

^1^Available online at: https://www.statista.com/statistics/1093256/novel-coronavirus-2019ncov-deaths-worldwide-by-country/ (accessed February 11, 2021).

^2^Available online at: https://en.unesco.org/covid19/educationresponse (accessed February 11, 2021).

^3^Available online at: https://afpa.org/content/uploads/2021/01/Position-de-la-SFP-AFPA-25-janvier-2021-002.pdf (accessed February 11, 2021).

^4^Available online at: https://www.cdc.gov/coronavirus/2019-ncov/prevent-getting-sick/cloth-face-cover-guidance.html (2019).

^5^Available online at: https://www.legifrance.gouv.fr/loda/id/JORFTEXT000042475143/2020-11-03/ (accessed February 11, 2021).

^6^Available online at: https://data.education.gouv.fr/explore/dataset/fr-en-sante-enfants-grande-section-et-cm2/export/ (accessed February 11, 2021).
