# Supplementary material for: Face Masks in Young Children During the COVID-19 Pandemic: Parents' and Pediatricians' Point of View
Source: Front Pediatr. 2021 Jun 23;9:676718. doi: 10.3389/fped.2021.676718 (PMC8260829; doi:10.3389/fped.2021.676718)
Supplement: Supplementary file 1 [file Data_Sheet_1.PDF]

Survey targeted to the parents

Survey targeted to the parents

Questions asked to parents
1. What is the age of your child for which you are answering?
2. Did you understand the reasons for making masks compulsory at school from the age of 6 years?
3. Why do you apply this measure for your child?
4. Have you explained to your child the reasons for the compulsory wearing of a mask at school?
5. Do you feel that your child has understood the reasons for wearing a mask at school?
6. Does your child wear the mask outside of school?
7. Since wearing the mask at school, has your child reported different symptoms? (several answers possible)
8. Since wearing the mask at school, have you noticed any changes in behavior? (several answers possible)
9. Overall, would you say that your child has become used to wearing the mask?
10. Can you ask your child directly « Are you embarrassed by the mask at school »?
11. Are you mother or father?
12. How old are you?
13. From which region are you from?

Survey targeted to the pediatricians

Questions asked to pediatricians
1. Do you agree with the compulsory wearing of a mask in school for a child over 6 years old?
2. During the consultation in your office, the wearing of a mask is spontaneously discussed by the parents?
3. During the consultation, you discuss the wearing of the mask with the child or his/her parents?
4. During the consultation, do parents spontaneously report to you any inconveniences related to the mask?
5. What drawbacks are most often cited?
6. Have you any consultations motivated by symptoms attributed to masks?
7. Have you received some requests for mask exemption certificate(s) seemingly unjustified ?
8. Most often, were you able to convince the child and/or his parents to continue wearing a mask
9. Have you received some requests for mask exemption certificate(s) seemingly justified ?
10. For which reason? (several answers possible)
11. Gender of pediatrician
12. Age of pediatrician
13. Region of pediatrician
